# Intestinal Anti-inflammatory Effects of Outer Membrane Vesicles from *Escherichia coli* Nissle 1917 in DSS-Experimental Colitis in Mice

**DOI:** 10.3389/fmicb.2017.01274

**Published:** 2017-07-11

**Authors:** María-José Fábrega, Alba Rodríguez-Nogales, José Garrido-Mesa, Francesca Algieri, Josefa Badía, Rosa Giménez, Julio Gálvez, Laura Baldomà

**Affiliations:** ^1^Departament de Bioquímica i Fisiologia, Facultat de Farmàcia i Ciències de l’Alimentació, Universitat de Barcelona, Institut de Biomedicina de la, Universitat de Barcelona Barcelona, Spain; ^2^Microbiota Intestinal, Institut de Recerca Sant Joan de Déu Esplugues de Llobregat, Spain; ^3^Centro de Investigación Biomédica en Red de Enfermedades Hepáticas y Digestivas, Department of Pharmacology, ibs.GRANADA, Center for Biomedical Research, University of Granada Granada, Spain

**Keywords:** probiotic, *Escherichia coli* Nissle 1917, outer membrane vesicles, DSS-experimental colitis, mouse model, immune modulation

## Abstract

*Escherichia coli* Nissle 1917 (EcN) is a probiotic strain with proven efficacy in inducing and maintaining remission of ulcerative colitis. However, the microbial factors that mediate these beneficial effects are not fully known. Gram-negative bacteria release outer membrane vesicles (OMVs) as a direct pathway for delivering selected bacterial proteins and active compounds to the host. In fact, vesicles released by gut microbiota are emerging as key players in signaling processes in the intestinal mucosa. In the present study, the dextran sodium sulfate (DSS)-induced colitis mouse model was used to investigate the potential of EcN OMVs to ameliorate mucosal injury and inflammation in the gut. The experimental protocol involved pre-treatment with OMVs for 10 days before DSS intake, and a 5-day recovery period. Oral administration of purified EcN OMVs (5 μg/day) significantly reduced DSS-induced weight loss and ameliorated clinical symptoms and histological scores. OMVs treatment counteracted altered expression of cytokines and markers of intestinal barrier function. This study shows for the first time that EcN OMVs can mediate the anti-inflammatory and barrier protection effects previously reported for this probiotic in experimental colitis. Remarkably, translation of probiotics to human healthcare requires knowledge of the molecular mechanisms involved in probiotic–host interactions. Thus, OMVs, as a non-replicative bacterial form, could be explored as a new probiotic-derived therapeutic approach, with even lower risk of adverse events than probiotic administration.

## Introduction

The term IBD mainly refers to UC and Crohn’s disease. These are chronic inflammatory disorders of the intestinal tract that may cause life-threatening complications and have an increasing incidence worldwide. They are multifactorial diseases, and although their etiology remains poorly understood, they involve a dysregulated immune response against commensal gut microbes in genetically susceptible individuals with impaired intestinal epithelial integrity (Zhang and Li, 2014). Moreover, several studies have reported that IBD patients have lower microbiota diversity than healthy subjects, and an altered intestinal microbiota balance, which is known as dysbiosis (Ott et al., 2004; Sokol et al., 2008; Manichanh et al., 2012).

Conventional IBD therapies are complex, and not all patients show complete beneficial effects when treated with any of the drugs currently available to manage these intestinal conditions, including salicylates, corticoids, immunosuppressants and biological agents (Ko and Auyeung, 2014). Since dysbiosis is common in IBD, various therapeutic approaches targeting the modulation of the gut microbiota have been explored (Qiao et al., 2016). In this context, many studies have evaluated the therapeutic potential of certain bacteria, including commensal and probiotic strains, to ameliorate IBD in clinical trials (Fedorak, 2010; Wasilewski et al., 2015) or in animal models of colitis (Ewaschuk et al., 2008; Garrido-Mesa et al., 2011; Shen et al., 2012; Kang et al., 2013; Martín et al., 2015; Souza et al., 2016). Overall, these studies have shown the ability of these bacteria to exert beneficial effects on parameters related with gut function, including improvement of gut permeability, reduction of inflammatory cytokine production and/or release, and amelioration of the histological alterations observed in these inflammatory conditions. In some cases, these effects may be mediated, at least in part, by bacterial secreted factors (Ewaschuk et al., 2008; Martín et al., 2015) or by released membrane vesicles (Shen et al., 2012; Kang et al., 2013).

The probiotic EcN positively affects gastrointestinal homeostasis and microbiota balance. In fact, various clinical trials have reported its therapeutic benefits in inducing and maintaining remission of UC (Scaldaferri et al., 2016). EcN shows similar efficacy to the aminosalicylate mesalazine (Losurdo et al., 2015), which supports its use in the treatment of human UC (Floch et al., 2011). Similarly, the intestinal anti-inflammatory effects of this probiotic have been reported in experimental models of colitis in mice and rats (Grabig et al., 2006; Ukena et al., 2007; Arribas et al., 2009; Garrido-Mesa et al., 2011; Souza et al., 2016). Several mechanisms have been reported to be involved in the beneficial effects of this probiotic in IBD, such as its ability to modulate the host immune response toward an anti-inflammatory balance (Trebichavsky et al., 2010). In addition, EcN reinforces the intestinal epithelial barrier by strengthening TJs between intestinal epithelial cells (Ukena et al., 2007; Zyrek et al., 2007; Hering et al., 2014) and increasing the expression of antimicrobial factors such as microcins (Sassone-Corsi et al., 2016) and β-defensin-2 (Schlee et al., 2007). It is important to note that these effects were mainly established when live probiotic suspensions were administered. There is little reported information describing the bacterial factors that could mediate these effects. In this context, membrane vesicles released by commensal bacteria are currently receiving great attention as key players in signaling processes in the intestinal mucosa (Kaparakis-Liaskos and Ferrero, 2015; Olsen and Amano, 2015). The release of bacterial vesicles provides a mechanism for the delivery of microbial proteins and active compounds directly to the host, thus avoiding direct intercellular contact. Recently, it has been reported that OMVs released by the probiotic EcN enter intestinal epithelial cells via clathrin-mediated endocytosis, and thereby can trigger host immune and defense responses (Cañas et al., 2016). In fact, *in vitro* and *ex vivo* studies have shown that EcN OMVs induce the expression of antimicrobial peptides and modulate the cytokine/chemokine response of gut epithelial and immune cells (Fábrega et al., 2016). In addition, in intestinal epithelial cell lines, these vesicles promote upregulation of the TJ-proteins ZO-1 and claudin-14, but down-regulation of claudin-2, thus reinforcing intestinal barrier functions and reducing gut permeability (Alvarez et al., 2016).

Probiotics are Generally Regarded As Safe (GRAS), yet some concerns about the potential risk associated with their use should be considered, especially in immunocompromised individuals and neonates. In conditions of altered intestinal barrier, the probiotic may cause invasive infections leading to sepsis (Ledoux et al., 2006; Bleich et al., 2008; Gronbach et al., 2010). In this context, the use of probiotic- derived bioactive factors, such as OMVs, could potentially have healing benefits, while avoiding the risks associated with the administration of live bacteria.

Based on previous *in vitro* studies (Alvarez et al., 2016; Fábrega et al., 2016) we hypothesized that EcN OMVs may contribute to the immunomodulatory and barrier strengthening effects of EcN in the gut. The aim of the present study was to evaluate the effects of OMVs isolated from EcN in the dextran sodium sulfate (DSS) model of mouse colitis, an experimental model that is widely used in preclinical assays to study new potential treatments for human IBD (Claes et al., 2011).

## Materials and Methods

### EcN Culture and OMVs Preparation

The probiotic strain EcN (serotype O6:K5:H1) was provided by Ardeypharm (GmbH, Herdecke, Germany). OMVs were isolated from culture supernatants of EcN grown overnight at 37°C in Luria-Bertani broth (LB) as previously described (Aguilera et al., 2014). Briefly, bacterial cells were pelleted by centrifugation at 10,000 × *g* for 20 min at 4°C. The supernatant was filtered through a 0.22 μm-pore-size filter (Millipore) to remove residual bacteria, concentrated by centrifugation in a Centricon Plus-70 filter device (Millipore) followed by an additional filtration step. OMVs were then recovered by centrifugation at 150,000 × *g* for 1 h at 4°C, washed and resuspended in sterile PBS. Aliquots were stored at -20°C. The sterility of samples was assessed on LB-agar plates. Protein concentration was measured using the method of (Lowry et al., 1951).

### Negative Staining and Transmission Electron Microscopy

Isolated OMVs were examined by transmission electron microscopy (TEM) after negative staining as described previously (Aguilera et al., 2014). A drop of OMV suspension was adsorbed for 2 min on Formvar/carbon coated-grids that were previously activated by UV light. Grids were washed with distilled water, stained with 2% uranyl acetate for 1 min, air dried and examined by TEM (Jeol, JEM 1010, Japan).

### Dextran Sodium Sulfate (DSS) Model of Mouse Colitis

**FIGURE 1:**
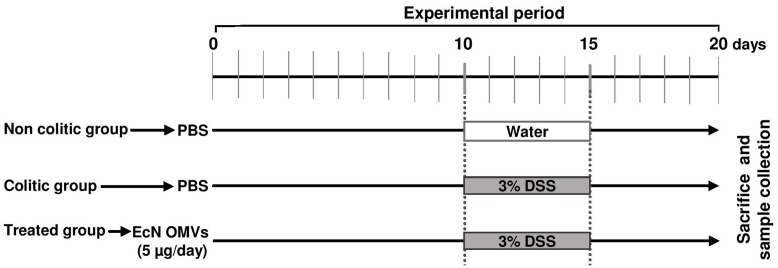
Experimental design to evaluate the potential of EcN OMVs to alleviate DSS-induced colitis in mice. The OMV-treated group daily received intragastrically 200 μl of PBS containing 5 μg of EcN OMVs over the 20-day experimental period, whereas the colitic group received 200 μl of PBS. The non-colitic control group was not challenged with DSS and received 200 μl of PBS over the entire experimental period.

All studies were carried out in accordance with the “Guide for the care and the Use of Laboratory Animals” as promulgated by the National Institute of Health. The protocol was approved by the Ethics Committee of the University of Granada (Ref. No. CEEA-2010-286). Male C57BL/6J mice (7- to 9-weeks-old; approximately 20 g) were obtained from Janvier (St Berthevin Cedex, France). Animals were randomly assigned to three groups: non-colitic (*n* = 6), colitic (*n* = 9) and OMVs-treated (*n* = 9). Mice were fed *ad libitum* during the entire experimental period (20 days) on AIN-93G growth purified diet, and animal body weight, as well as food and water intake, were evaluated daily. The experimental design is shown in **Figure 1**. Every day throughout the experimental period (20 days), the two reference groups (non-colitic and colitic) received PBS solution (200 μl), and the treated group received EcN OMVs (5 μg in 200 μl of PBS) by means of an oesophageal catheter. Ten days after starting the experiment, colitis was induced by adding DSS (36–50 kDa, MP Biomedical, Scarborough, ON, United States) to the drinking water at a final concentration of 3% (Mähler et al., 1998). This treatment was applied to all groups for 5 days, except for mice from the reference non-colitic group, which drank tap water throughout the experiment. During this DSS-induced colitis period, the OMVs-treated group was daily administered with EcN OMVs (5 μg/mouse in 200 μl PBS). After colitis damage, 200 μl of PBS were administered daily to all groups except for the OMVs-treated model, which continued with the OMVs treatment for five additional days. All mice were sacrificed 20 days after beginning of the experiment. Animals were previously anesthetized with ketamine (100 mg/kg) and xylazine (10 mg/kg). After starting the DSS intake, animal body weight, the presence of gross blood in the feces and stool consistency were individually evaluated daily (by an observer who was unaware of the treatment). Each parameter was assigned a score according to the criteria proposed previously by (Cooper et al., 1993) and used to calculate an average daily DAI (**Table 1**).

**Table 1:** Scoring index for disease activity.

Score	Weight loss (%)	Stool consistency	Rectal bleeding
0	None	Normal	Normal
1	1–5		
2	5–10	Loose stools	
3	10–20		
4	>20	Diarrhea	Gross bleeding


*Disease Activity Index was calculated by adding combined scores for weight loss, stool consistency and rectal bleeding, and dividing the resulting global score by three. Adapted from Cooper et al. (1993).*

Once mice were sacrificed, the colon was excised from anus to caecum, emptied and washed with PBS before being measured and weighed. Representative specimens (0.5 cm length containing all wall layers) were taken from the distal inflamed region and fixed in 4% buffered formaldehyde for histological analysis. Other fragments were placed in RNA later stabilization reagent (Qiagen GmbH, Hilden, Germany) and stored at -80°C until RNA extraction. The remaining colonic tissue was subsequently sectioned in different longitudinal fragments to be used for biochemical determinations.

### Colonic Organ Culture and ELISA Assays

Small fragments of colonic tissue were weighted, cut and then cultured in DMEM (Gibco, Grand Island, NY, United States) supplemented with 10% (v/v) of heat inactivated fetal bovine serum, 1% penicillin/streptomycin, 1% amphotericin, 2% glutamine and 0.45% glucose and incubated at 37°C in a 5% CO_2_ atmosphere. After 24-h culture, the supernatants were collected, centrifuged and stored at -80°C until assay. IL-1β, IL-6 and IL-10 levels were measured using enzyme-linked immunosorbent assay (ELISA) kits (R&D Systems, Minneapolis, MN, United States) according to the manufacturer’s instructions. The results were expressed as pg/mg protein.

### Histological Studies

The colonic samples that were fixed in formaldehyde were paraffin-embedded, sectioned (5 μm) at different levels, and stained with haematoxylin and eosin. Tissues were reviewed in a blinded fashion and the histological damage was assessed according to a previously validated intestinal histologic inflammatory score (Camuesco et al., 2012). This score takes into account the presence of ulceration, infiltration, oedema and the condition of crypts. After evaluation of these features a score ranging from 0 (healthy tissue) to 3 or 4 (severe damage), depending on the item, was assigned to each one. For each sample, the total score was calculated from the sum of each item’s score.

### Gene Expression Analysis in Colonic Samples by RT-qPCR

Tissue was homogenized in 1 ml of QIAzol using a Precellys 24 homogenizer (Bertin Technologies, Montigny-Le bretonneux, France). Total RNA was isolated using RNeasy Mini Kit (Qiagen, Hilden, Germany) following the manufacturer’s protocol. Purity and RNA concentration were measured by the absorbance ratio at 260 and 280 in a Thermo Scientific Nano Drop TM 2000 Spectrophotometer. RNA integrity was verified by visualization of 28S and 18S rRNAs after 1% agarose/formaldehyde gel electrophoresis. RNA (2 μg) was reverse transcribed using oligo (dT) primers (Promega, Southampton, United Kingdom). The resulting cDNA (20 ng) was amplified on optical grade 48-well plates in an Eco^TM^ Real Time PCR System (Illumina, San Diego, CA, United States), using the KAPA Sybr Fast qPCR Master Mix (Kapa Biosystems, Inc., Wilmington, MA, United States) and specific primers for each gene (**Table 2**). The 2^-ΔΔCt^ method was used to normalize expression results. The values of the housekeeping glyceraldehyde-3-phosphate dehydrogenase (GAPDH) gene were used to normalize the values obtained for each of the genes under study. Relative gene expression was calculated by means of the ΔΔCt formula and expressed as fold-change compared with the non-colitic control.

**Table 2:** Primer sequences used for quantitative RT-PCR.

Gene	Sequence (5′-3′)	Annealing T (°C)	Gene accession number
GAPDH	FW: CCATCACCATCTTCCAGGAG	60	NM_008084
	RV: CCTGCTTCACCACCTTCTTG		
IL-1β	FW: TGATGAGAATGACCTCTTCT	60	NM_008361.4
	RV: CTTCTTCAAAGATGAAGGAAA		
IL-6	FW: TAGTCCTTCCTACCCCAATTTCC	60	NM_031168.2
	RV: TTGGTCCTTAGCCACTCCTTCC		
IL-10	FW: TCCTTAATGCAGGACTTTAAGGG	56	NM_010548.2
	RV: GGTCTTGGAGCTTATTAAAAT		
IL-12	FW: CCTGGGTGAGCCGACAGAAGC	60	NM_001159424
	RW: CCACTCCTGGAACCTAAGCAC		
IL-17	FW: GCTCCAGAAGGCCCTCAGACTACC	60	NM_010552.3
	RV: CTTCCCTCCGCATTGACACAGC		
TNF-α	FW: AACTAGTGGTGCCAGCCGAT	60	NM_013693.3
	RV: CTTCACAGAGCAATGACTCC		
INF-γ	FW: GAACTGGCAAAAGGATGGTGA	60	NM_008337.4
	RV: TGTGGGTTGTTGACCTCAAAC		
MIP-2	FW: AGTTAGCCTTGCCTTTGTTCAG	57	NM_009140.2
	RV: CAGTGAGCTGCGCTGTCCAATG		
MMP-2	FW: TGCCGGCACCACTGAGGACTAC	56	XM_006530751.2
	RV: GGGCTGCCACGAGGAACA		
MMP-9	FW: TGGGGGCAACTCGGC	60	NM_013599.4
	RV: GGAATGATCTAAGCCCAG		
iNOS	FW: GTTGAAGACTGAGACTCTGG	67	NM_010927.4
	RV: ACTAGGCTACTCCGTGGA		
COX-2	FW: GGGTTGCTGGGGGAAGAAATG	60	NM_011198.4
	RV: GGTGGCTGTTTTGGTAGGCTG		
TFF-3	FW: CCTGGTTGCTGGGTCCTCTG	60	NM_011575.2
	RV: GCCACGGTTGTTACACTGCTC		
Occludin	FW: ACGGACCCTGACCACTATGA	56	NM_008756.2
	RV: TCAGCAGCAGCCATGTACTC		
ZO-1	FW: GGGGCCTACACTGATCAAGA	56	XM_006540782
	RV: TGGAGATGAGGCTTCTGCTT		


### SDS–PAGE and Western Blot Analysis

Protein concentration of colonic samples was measured using a BCA Protein Assay Kit. The intestinal inducible isoform of nitric oxide synthase (iNOS) was analyzed by Western blot. Samples (50 μg protein) were mixed with SDS–PAGE sample buffer, boiled for 5 min at 95°C and electrophoresed on 6% SDS–PAGE gel. Proteins were transferred to a HyBond-P polyvinylidene difluoride membrane using a Bio-Rad Mini Transblot apparatus. The membrane was blocked in PBS-0.05% Tween-20 and 5% skimmed milk (blocking solution) for 1 h at room temperature, and then incubated with specific antibodies against iNOS (Transduction Laboratories, Becton Dickinson Biosciences, Madrid, Spain), 1:2,000 dilution in blocking solution, for 16 h at 4°C, followed by incubation with peroxidase-conjugated anti-rabbit IgG antibody (1:3,000) for 1 h. The protein-antibody complex was visualized using the ECL Plus Western blotting detection system (Amersham Pharmacia Biotech). Control of protein loading and transfer was conducted by detection of the β-actin levels.

### Statistics

Statistical analysis was performed using SPSS version 20.0 software (SPSS, Inc.) and GraphPad Prism version 6.0 (GraphPad Software Inc., La Jolla, CA, United States), and data were expressed as mean ± SEM. Differences between more than two groups were assessed using one-way ANOVA followed by Tukey’s test. Significant differences were established at *P* ≤ 0.05.

## Results

### EcN OMVs Improve Clinical Signs and Colonic Histology in DSS-Induced Mouse Colitis

**FIGURE 2:**
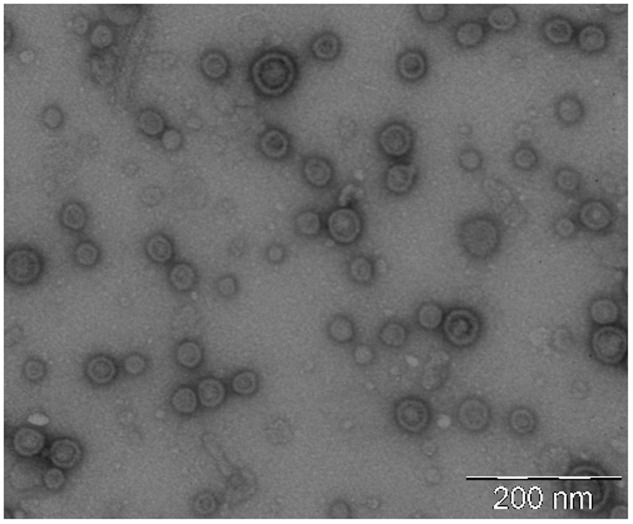
Negative staining electron microscopy of isolated EcN OMVs. Vesicles were isolated from 1-liter LB culture of EcN and resuspended in a final volume of 0.2 ml PBS. A representative image of OMV samples (1:20 dilution) is shown. Scale bar: 200 nm.

OMVs were isolated from cell-free culture supernatants and evaluated by negative stain-TEM. Images showed spherical vesicles ranging in size from approximately 20–60 nm in diameter (**Figure 2**).

**FIGURE 3:**
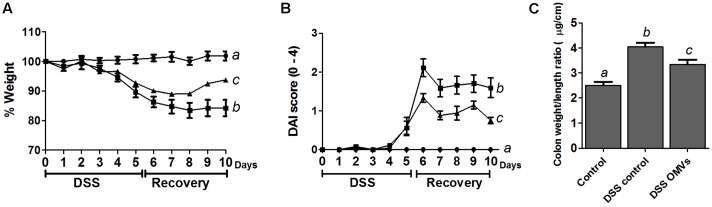
EcN OMVs treatment improves clinical signs of DSS-induced colitis in mice. After the 10-day pre-treatment period, mice received 3% DSS in drinking water for 5 days. Mice were sacrificed 5 days later. Mice from the OMVs-treated group (triangles; *n* = 9) were administered with EcN OMVs (5 μg/day) throughout the experiment, whereas the two other experimental groups, colitic DSS control (squares; *n* = 9) and non-colitic control (circles; *n* = 6), received PBS instead. **(A)** Weight evolution in animals over the DSS-treatment and the recovery period. Values are presented as percentage of the body weight at the beginning of DSS intake (day 0). **(B)** DAI score of each experimental group from the beginning of the induction of colitis by DSS treatment (day 0) until sacrifice (day 10), calculated as described in the Section “Materials and Methods.” **(C)** Colon weight/length ratio calculated following resection. Data are expressed as means ± SEM. Different letters indicate significant differences between groups (*P* < 0.05).

The *in vivo* protective effects of EcN OMVs were evaluated in male C57BL/6J mice treated with 3% DSS following the protocol outlined in **Figure 1**, which involves pre-treatment with OMVs for 10 days before DSS intake, and a 5-day recovery period. The administration of EcN OMVs for 10 days before DSS intake did not result in significant modifications in body weight in comparison with non-treated mice. Body weight increase was around 15–17% in all groups. When DSS was orally administered to mice, the intestinal inflammatory status was induced, and characterized in the control colitic group (DSS control) by marked body weight loss (**Figure 3A**) and diarrhea with bleeding feces, which resulted in an increased DAI score in this group from day 5 (**Figure 3B**). The administration of EcN OMVs to colitic mice (DSS-OMVs) ameliorated body weight loss (**Figure 3A**) and notably reduced the DAI score from day 6 after DSS intake in comparison with the control colitic group. Statistically significant differences were obtained on the last day of the study (**Figure 3B**). Once mice had been sacrificed, macroscopic evaluation of colonic segments confirmed the beneficial impact of EcN OMVs on the inflammatory process. In fact, colon length is inversely associated with the severity of DSS-induced colitis (Pandurangan et al., 2015), and the colonic weight/length ratio is widely used as an indicator of the colonic oedema that typically occurs in experimental colitis (Hou et al., 2014). The colonic weight/length ratio values were significantly higher in the colitic group than in the OMVs-treated group (**Figure 3C**), thus revealing that EcN OMVs intake attenuated the colonic oedema associated with DSS-induced intestinal inflammation in mice.

**FIGURE 4:**
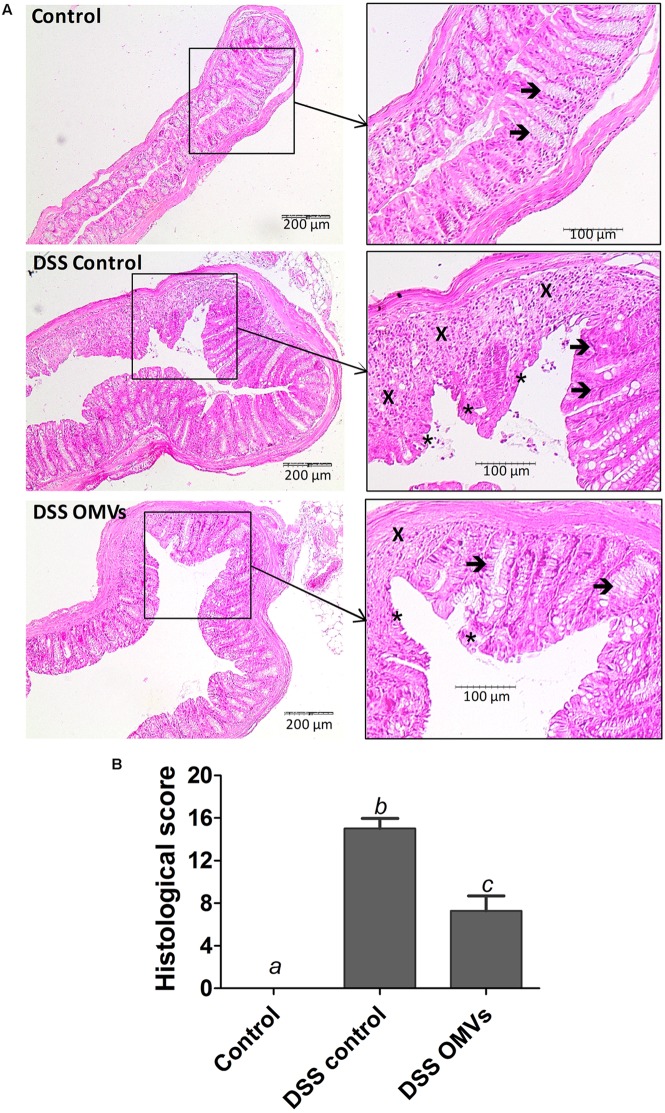
EcN OMVs treatment promotes recovery of DSS-induced intestinal injury and inflammation in mice. On day 20, colons were excised and processed for microscopic analysis. **(A)** Histological images of colonic tissue stained with haematoxylin and eosin showing the effect of EcN OMVs on DSS-induced colitis. Representative images of each experimental group are shown: Control, DSS control and DSS OMVs. In Control, images show the normal appearance of the intact mucosa containing the crypts with goblet cells plenty of their mucin content (arrows). In DSS control, images show changes in the mucosa with areas of ulceration on the epithelial layer (asterisks), reduction of goblet cells with depletion of their mucin content (arrows) and intense inflammatory cell infiltrate (cross). In DSS OMVs, an improvement of the colonic histology is observed, with reduced area of ulceration, mostly in process of recovery (asterisks), presence of goblet cells replenished with their mucin content (arrow) and reduced inflammatory cell infiltrate (cross). **(B)** Histological scores calculated after microscopic analyses of longitudinal colon sections as described in the Section “Materials and Methods.” Results are expressed as mean ± SEM. Control group (*n* = 6), DSS control group (*n* = 9), DSS OMVs group (*n* = 9). Different letters indicate significant differences between groups (*P* < 0.05).

Microscopic evaluation of the colonic samples also evidenced the intestinal anti-inflammatory effects of EcN OMVs on DSS-induced colitis. Intestinal tissue from the control colitic group revealed common characteristics that were previously reported for this experimental model of colitis (Algieri et al., 2014), including mucosal ulceration, with loss of the normal crypt structure and intense goblet cell depletion, together with inflammatory cell infiltration and oedema (**Figure 4A**). Evaluation of colonic damage by the scoring method described above yielded high histological scores in colitic mice (DSS control group). In contrast, the colitic group treated with EcN OMVs showed recovery from the inflammatory process with an improvement in mucosal barrier integrity, as evidenced by a smaller ulceration surface and the presence of goblet cells replenished with their mucin content. In addition, samples from mice treated with OMVs showed lower inflammatory cell infiltration and tissue oedema than samples from the control colitic group. Consistently, microscopic evaluation of colonic damage in the OMV-treated colitic mice resulted in significant improvement in the histologic scores (*P* < 0.05 vs. control colitic group) (**Figure 4B**).

### Intestinal Anti-inflammatory Effects of EcN OMVs Are Associated with an Improvement in the Altered Immune Response in DSS-Induced Mouse Colitis

**FIGURE 5:**
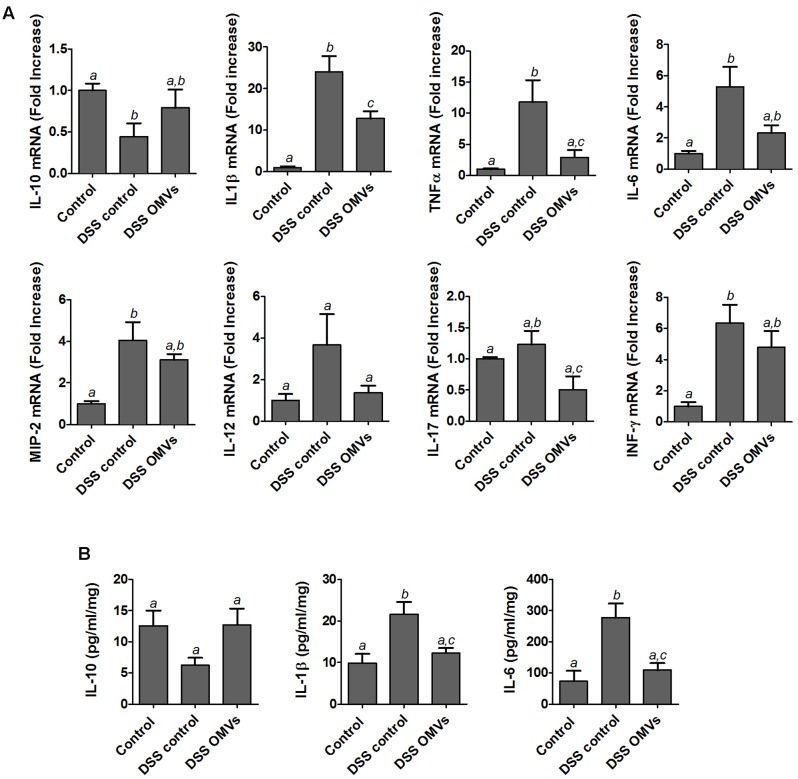
Effects of EcN OMVs treatment on colonic cytokine expression in DSS colitic mice. **(A)** Relative mRNA levels of the indicated cytokines measured by RT-qPCR in colonic tissue. Data are presented as fold-change compared to the non-colitic control group (expression value set to 1). **(B)** IL-10, IL-1β and IL-6 protein levels measured by ELISA in culture supernatants of colonic fragments from mice of each experimental group incubated for 24 h in complete DMEM medium as described in the Section “Materials and Methods.” Data are expressed as mean ± SEM. Control group (*n* = 6), DSS control group (*n* = 9), DSS OMVs group (*n* = 9). Different letters indicate significant differences between groups (*P* < 0.05).

It is well-known that DSS-induced colonic inflammation is associated with an altered immune response (Xu et al., 2014). Consistently, results from the RT-qPCR analysis performed in the present study revealed that mice from the colitic group showed significant higher mRNA expression of the pro-inflammatory cytokines IL-1β, TNF-α, IL-6, MIP-2 and INFγ than the non-colitic group. A trend of higher expression of cytokines IL-12 and IL-17 was also observed in the colitic group but the values did not reach statistical significance when compared with non-colitic mice. The treatment of colitic mice with EcN OMVs decreased the expression of all pro-inflammatory cytokines assayed. The results were statistically significant for IL-1β, TNF-α and IL-17 in comparison with control colitic mice (**Figure 5A**). Moreover, the colonic inflammatory process induced by DSS was associated with lower expression of the anti-inflammatory cytokine IL-10, which was significantly counteracted by administration of EcN OMVs (**Figure 5A**).

The observed changes in colonic mRNA expression correlated well with the protein levels, as evidenced from the quantitative ELISA results of three representative cytokines (IL-10, IL-6 and IL-1β) (**Figure 5B**). Levels of the pro-inflammatory cytokines IL-6 and IL-1β were significantly lower in the colitic group treated with EcN OMVs than in the corresponding control group (DSS control). Administration of EcN OMVs counterbalanced the DSS-mediated reduction in the colonic levels of the anti-inflammatory cytokine IL-10, although these results did not reach statistical significance, probably due to variability in the data (**Figure 5B**).

**FIGURE 6:**
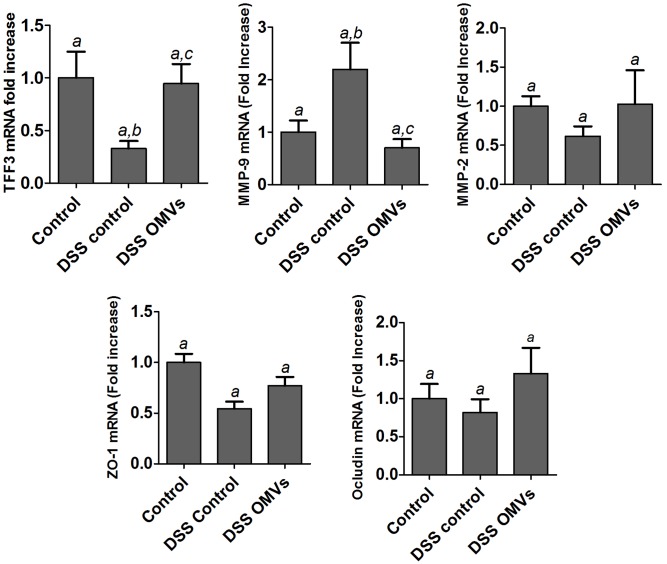
Effects of EcN OMVs treatment on colonic expression of markers of intestinal barrier function in DSS colitic mice. Relative mRNA levels of TFF-3, MMP-9, MMP-2, ZO-1 and occludin were measured by RT-qPCR in colonic tissue. Data are presented as fold-change compared to the non-colitic control group. Control group (*n* = 6), DSS control group (*n* = 9), DSS OMVs group (*n* = 9). Data are presented as mean ± SEM. Different letters indicate significant differences between groups (*P* < 0.05).

The beneficial effects of EcN OMVs were also associated with the recovery of a marker of intestinal barrier function, the intestinal TFF-3, whose expression is downregulated in inflamed colonic tissue after DSS administration to mice. It is interesting to note that EcN OMVs restored the mRNA levels of TFF-3 to values similar to those found in the non-colitic control group (**Figure 6**). Treatment with EcN OMVs did not counteract DSS-induced downregulation of ZO-1, but tended to increase occludin mRNA levels in DSS-treated mice, although data did not reach statistical significance (**Figure 6**). In the context of tissue remodeling, we also measured the mRNA expression of two relevant MMPs, MMP-9 and MMP-2, which have opposing roles. MMP-9 promotes tissue injury in colitic mice, whereas MMP-2 has a protective role and contributes to the maintenance of gut barrier function (Garg et al., 2009). Accordingly, mice in the colitic group showed greater expression of MMP-9 and lower expression of MMP-2 than non-colitic mice. Both DSS-mediated effects were countered by treatment with EcN OMVs, but only the reduction in colonic MMP-9 mRNA levels was statistically significant (**Figure 6**). Consequently, the effects exerted by EcN OMVs on the altered expression of MMPs during colitis can contribute to their ability to protect against tissue damage induced by DSS.

**FIGURE 7:**
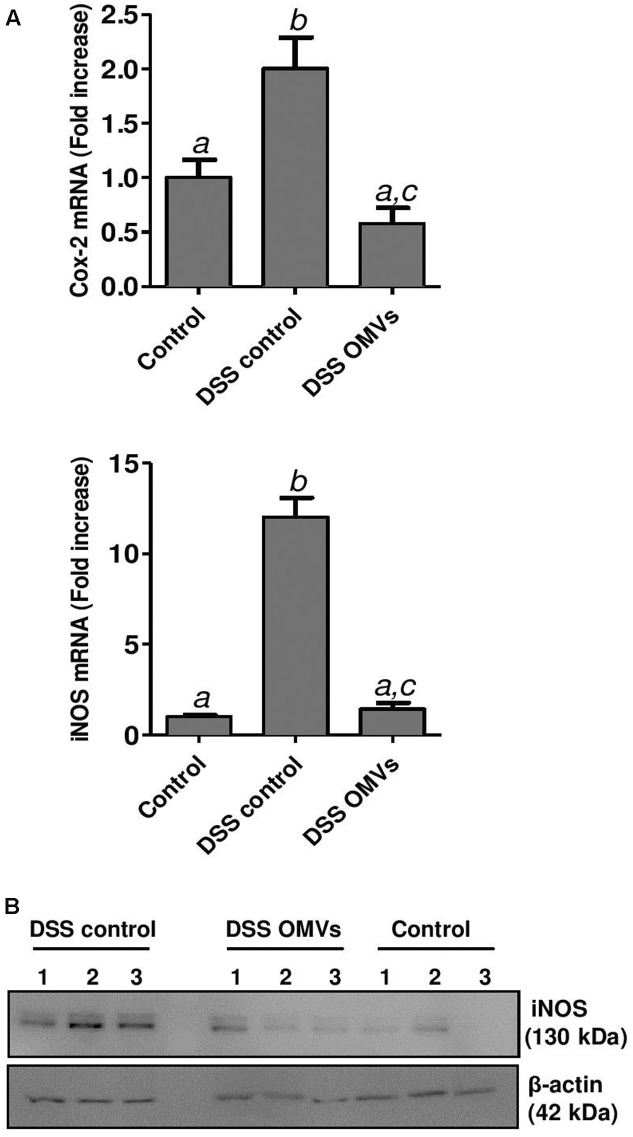
Effects of EcN OMVs treatment on colonic COX-2 and iNOS expression. **(A)** Relative mRNA levels of COX-2 and iNOS measured by RT-qPCR in colonic tissue. Data are presented as fold-change compared to the non-colitic control group. Different letters indicate significant differences between groups (*P* < 0.05). **(B)** Western blot analysis of iNOS levels in colonic fragments collected from three mice (lines 1, 2, 3) of each group. Anti-β-actin antibody was used as internal control. Representative images are shown.

In addition, RT-qPCR analysis was performed for the inflammatory enzymes COX-2 and inducible iNOS, known to be upregulated in DSS-induced colitis. Administration of EcN OMVs to colitic mice significantly reduced the expression of both enzymes, which were highly expressed in the colonic tissue of untreated colitic mice (**Figure 7A**). Western blotting of iNOS confirmed the compensatory effect mediated by EcN OMVs at protein level (**Figure 7B**).

## Discussion

The etiology of IBD has not been completely elucidated to date. However, it has been proposed that intestinal dysbiosis is crucial, since it can promote an inappropriate immune response in genetically susceptible individuals, leading to the inflammatory status that characterizes these intestinal conditions (Becker et al., 2015). Accordingly, restoration of intestinal microbiota composition through the administration of probiotics could have beneficial effects against the inflammatory response, as has been shown both in experimental models and in human IBD (Claes et al., 2011; Bellaguarda and Chang, 2015). EcN is one of the probiotics reported to have a beneficial effect in IBD, and plays a prominent role in therapy for this group of intestinal conditions in humans. In fact, the 2011 update of the American Recommendations for Probiotic Use gave very strong “A” recommendations for its use to maintain remission in human UC (Floch et al., 2011). The therapeutic efficacy of EcN has been more recently supported in a systematic review and meta-analysis (Losurdo et al., 2015). Among the mechanisms that might be involved in the beneficial effects exerted by EcN, its immunomodulatory properties could play a key role. In the gut, the probiotic can counteract altered cytokine production by immune cells (Sturm et al., 2005; Guzy et al., 2008) and restore damaged epithelium through the modulation of TJ and zonula occludens proteins (Ukena et al., 2007; Zyrek et al., 2007). Although direct interaction between the bacteria EcN and the immune or epithelial cells could be responsible for the observed effects, other mechanisms involving the production and release of secreted bacterial factors cannot be ruled out. Among these secreted factors, membrane vesicles play a key role in bacteria–host communication, allowing the delivery of effector molecules upon interaction and internalization into the host cells. In fact, OMVs enclose many of the biological components that can be found in the bacterium and, specifically, ligands of pattern recognition receptors such as LPS (TLR-4 ligand), peptidoglycan (NOD-1/NOD-2 ligand) or DNA (TLR-9 ligand), among others. These receptors, which are expressed in epithelial and immune cells, are key components of innate immunity as they sense gut microbes and trigger appropriate immune responses. Therefore, bacteria-released OMVs can mediate most of the effects of the bacterium. Remarkably, OMVs as non-replicative bacterial forms further reduce the low risk of adverse events associated with the administration of the probiotic (Bleich et al., 2008; Gronbach et al., 2010; Olier et al., 2012).

Previous studies performed in human cell lines and human colonic explants have shown the immunomodulatory and barrier strengthening potential of EcN OMVs (Alvarez et al., 2016; Fábrega et al., 2016). Since orally administered live EcN suspensions ameliorate DSS-induced colitis in mice (Grabig et al., 2006; Garrido-Mesa et al., 2011; Olier et al., 2012), we sought to explore whether EcN OMVs could prevent colonic damage in the same experimental model of intestinal inflammation. This is a well-established animal model that resembles human UC, and it has been validated for the translation of mouse data to humans (Jiminez et al., 2015).

The present study demonstrates that oral administration of OMVs isolated from EcN has intestinal anti-inflammatory properties in the DSS experimental model of colitis, as previously reported when a suspension of the viable probiotic was administered (Garrido-Mesa et al., 2011). The beneficial effects of EcN OMVs were already observed during the course of the experiment. In fact, reduction in weight loss and DAI evolution in mice treated with OMVs, compared to the control colitic group, clearly evidenced amelioration of the impact of the colitic process and improvement in the health status of mice.

Likewise, OMVs administration to colitic mice significantly reduced colonic damage. This was shown macroscopically by a significant reduction in the colonic weight/length ratio, which indicated amelioration of the tissue oedema that characterizes this colonic inflammatory process, and microscopically with recovery of the altered tissue histology associated with DSS colitis. The OMV-mediated anti-inflammatory effect was clearly associated with an improvement in the altered immune response accompanying DSS-induced colonic damage, thus mimicking the immunomodulatory properties described for this probiotic both *in vivo* and *in vitro* (Kruis et al., 2004; Grabig et al., 2006; Arribas et al., 2009; Garrido-Mesa et al., 2011; Scaldaferri et al., 2016). It is known that the acute mucosal inflammation induced by DSS results in activation of several cells involved in the intestinal innate immune response, including epithelial cells, macrophages and dendritic cells, leading to upregulated expression and/or production of pro-inflammatory cytokines, such as IL-1β, TNF-α and IL-6. This subsequently facilitates the sustained inflammatory response associated with an imbalance in Th1/Th17 and Treg cell responses, which results in increased expression and/or production of other cytokines such as IFN-γ, IL-12 (Th1) and IL-17 (Th17), together with a reduction in the anti-inflammatory cytokine IL-10 (Treg) (Neurath, 2014). Our results show that administration of EcN OMVs ameliorates the altered cytokine profile observed in colitic mice treated with DSS.

Different mechanisms could be involved in these immunomodulatory properties. Of particular significance is the ability to restore intestinal epithelial integrity. In this context, it has been reported that the intestinal anti-inflammatory properties of EcN are related to its ability to reinforce TJs of intestinal epithelial cells, thus counteracting the altered permeability status in the intestinal epithelium that characterizes intestinal inflammation (Ukena et al., 2007). Notably, impairment of the epithelial barrier function has been proposed as one of the first events to occur in intestinal inflammation. This facilitates the access of antigens from the intestinal lumen and triggers the exacerbated immune response. Consequently, rapid promotion of mucosal healing has been considered crucial in the management of intestinal inflammation (Dave and Loftus, 2012). Under conditions of intact epithelial barrier EcN OMVs (100 μg/ml) upregulate expression of claudin-14 and ZO-1 in T-84 and Caco-2 monolayers (Alvarez et al., 2016). However, oral administration of EcN OMVs does not prevent DSS-induced downregulation of ZO-1 mRNA. Thus, the effects previously observed in *in vitro* models of intact epithelial barrier are not appreciated in the *in vivo* model of experimental colitis. This suggests that different regulatory mechanisms could be activated by EcN OMVs in the presence of highly expressed inflammatory mediators. Notably, ZO-1 is a target of post-translational modifications that regulate the intracellular fate of this protein. Reinforcement of the epithelial barrier can also be triggered by stimuli that promote ZO-1 location at the cell boundaries, and hence its association with TJ structures. In this context, it has been described that TFF-3 promotes redistribution of ZO-1 from the cytoplasmic compartment to the intercellular junctions in Caco-2 cell monolayers, which results in an increased level of co-localisation with occludin. These regulatory effects were not accompanied by changes in ZO-1 or occludin protein levels (Buda et al., 2012). Expression of TFF-3 is downregulated in intestinal inflammation (Lozano-Pérez et al., 2014), as observed in the present study. In connection with this, here we show that EcN OMVs increase colonic expression of this bioactive peptide involved in epithelial protection and repair (Podolsky et al., 2009), and preserve the damaged colonic cytoarchitecture of the mucosa in colitic mice. In addition to beneficial effects on epithelial integrity restoration, increased expression of TFF-3 can help to ameliorate the altered immune response exerted by EcN OMVs in experimental colitis, since overexpression of this peptide has been reported to abolish the IL-1β-induced upregulation of the pro-inflammatory cytokines IL-6, IL-8 and TNFα (Lin et al., 2013). Another plausible mechanism exploited by EcN OMVs to protect intestinal barrier function in colitic mice is downregulation of MMP-9 expression. Upregulation of this gelatinase contributes to the pathogenesis of IBD by disrupting TJs between intestinal epithelial cells, leading to increased intestinal permeability and the subsequent pro-inflammatory effects (Nighot et al., 2015). Moreover, treatment of colitic mice with EcN OMVs tends to increase, or at least preserve, MMP-2 expression, thus protecting against DSS-associated intestinal barrier loss (Garg et al., 2009).

Other markers of inflammation and tissue damage that are over-expressed in DSS-induced colitis are COX-2 and iNOS (Kolios et al., 2004). These enzymes are in fact defense mechanisms against the injury, inflammation and infection that occur in pathological conditions, and are highly expressed at damaged sites. Increases in COX-2 levels are seen in chronic intestinal inflammation, which is one of the risk factors for colorectal cancer (Wang and DuBois, 2010). Expression of iNOS is induced by certain pro-inflammatory cytokines such as TNF-α or IFN-γ, as well as by bacterial products. Although increased NO production through upregulation of iNOS is part of the intestinal antibacterial response, excess NO has been associated with intestinal inflammation in IBD patients and attributed to the immune dysregulation that occurs in these intestinal disorders (Kolios et al., 2004). In fact, elevated iNOS expression has been demonstrated in clinical IBD, caused mainly by infiltrating macrophages in intestinal mucosa (Rachmilewitz et al., 1995; Palatka et al., 2005). In addition, excessive NO production mainly through iNOS has been correlated with tissue injury and mucosal lesions observed in both human IBD and DSS-induced colitic mice. Consequently, probiotics or natural compounds with proven efficacy in attenuating colitis in DSS-treated mice reduce iNOS expression (Camuesco et al., 2004; Garrido-Mesa et al., 2015; Ren et al., 2015; Senol et al., 2015; Utrilla et al., 2015). EcN is among these probiotics (Garrido-Mesa et al., 2011). Here we show for the first time the ability of OMVs from a probiotic strain, specifically EcN, to counteract DSS-induced expression of both iNOS and COX-2. This correlates with reduced expression of the pro-inflammatory cytokines TNF-α and IFN-γ, and lower inflammatory cell infiltration in OMVs-treated mice.

At present, the molecules in EcN OMVs responsible for the anti-inflammatory and/or barrier protective effects remain unknown. Olier et al. (2012) reported that the genotoxic polyketide colibactin is required for the *in vivo* anti-inflammatory effects of EcN. Moreover, deficiency in colibactin biosynthesis leads to exacerbation of colitis severity in DSS-treated mice. These facts lead authors to considered colibactin as an immunomodulin. Whether this bacterial effector is exported and delivered into the host cells remains elusive. As EcN OMVs exert anti-inflammatory effects in experimental colitis, we may speculate that colibactin could be secreted through OMVs, at least in part. However, the contribution of other specific probiotic OMV-associated factors cannot be ruled out. Regarding mediators of intestinal barrier protection, it has been shown that the secreted protein TcpC contributes to the *in vitro* strengthening ability of EcN when the epithelial barrier is intact (Hering et al., 2014). However, the effect of TcpC is not associated with OMVs (Alvarez et al., 2016). Thus, OMV-linked factors other than TcpC should contribute to the modulation of mucosal healing markers like TFF-3 and MMP-9, observed in this experimental murine colitis model.

## Conclusion

Recently acquired knowledge shows that microbiota plays an important role in the pathogenesis of IBD; dysbiosis is a common feature of these inflammatory conditions. Nowadays, potential clinical applications of probiotics are being explored to restore microbiota imbalances and take advantage of their health-promoting capacities. However, the translation of probiotics or microbiota-based drugs to human health-care requires deep knowledge of the molecular mechanisms involved in microbiota–host interactions (Jia et al., 2008; Shanahan, 2011). In this regard, bacterial vesicles are key players in signaling processes in the intestinal mucosa, as they act as a secretion and delivery pathway for selected bacterial proteins and active compounds directly to the host cells. Among probiotics with promising results in IBD clinical assays and experimental colitis models, EcN has beneficial effects on the remission of UC. Our study proves that oral administration of OMVs isolated from this probiotic has intestinal anti-inflammatory effects in the DSS experimental model of colitis, similarly as previously reported for the administration of viable probiotic suspensions. Therefore, OMVs could represent a safe probiotic-derived strategy targeting intestinal inflammatory processes as they are free from bacteria and can elicit the same effect on gastrointestinal health as the probiotic itself.

## Author Contributions

LB, JG, JB and RG contributed to the conception and design of the study. M-JF and AR-N were the responsible to perform all the experiments and analysis of data. JG, JG-M, and FA contributed to the histological studies. JG, LB, JB, and RG supervised the work, interpreted the data and wrote the drafted manuscript. All the authors revised the manuscript and approved the final version.

## Conflict of Interest Statement

The authors declare that the research was conducted in the absence of any commercial or financial relationships that could be construed as a potential conflict of interest.

## Abbreviations

Cox-2: cyclooxygenase 2
DAI: disease activity index
DSS: dextran sodium sulfate
EcN: *Escherichia coli* Nissle 1917
GAPDH: glyceraldehyde-3-phosphate dehydrogenase
hBD-2: human β-defensin
IBD: inflammatory bowel diseases
iNOS: inducible nitric oxide synthase
LB: Luria-Bertani broth
LPS: lipopolysaccharide
MIP-2: macrophage inflammatory protein 2
MMPs: matrix metalloproteinases
NO: nitric oxide
OMVs: outer membrane vesicles
PAGE: polyacrylamide gel electrophoresis
PBMCs: peripheral blood mononuclear cells
PBS: phosphate buffered saline
PRRs: pattern-recognition receptors
SDS: sodium dodecyl sulfate
TEM: transmission electron microscopy
TFF-3: trefoil factor 3
TJ: tight junction
TLRs: toll-like receptors
TNF-α: tumor necrosis factor-α
Treg: regulatory T cells
UC: ulcerative colitis
ZO-1: zonula occludens-1
